# Improving Palliative Cancer Care

**DOI:** 10.6004/jadpro.2014.5.5.3

**Published:** 2014-09-01

**Authors:** Catherine Del Ferraro, Betty Ferrell, Carin Van Zyl, Bonnie Freeman, Linda Klein

**Affiliations:** City of Hope, Duarte, California

## Abstract

Over a decade ago, the Institute of Medicine (IOM) presented *Ensuring Quality Cancer Care* in the United States, with recommendations for change (IOM, 1999). However, barriers to integrating palliative care (PC) to achieve high-quality care in cancer still remain. As novel therapeutic agents evolve, patients are living longer, and advanced cancer is now considered a chronic illness. In addition to complex symptom concerns, patients and family caregivers are burdened with psychological, social, and spiritual distress. Furthermore, data show that PC continues to be underutilized and inaccessible, and current innovative models of integrating PC into standard cancer care lack uniformity. The aim of this article is to address the existing barriers in implementing PC into our cancer care delivery system and discuss how the oncology advanced practice nurse plays an essential role in providing high-quality cancer care. We also review the IOM recommendations; highlight the work done by the National Consensus Project in promoting quality PC; and discuss a National Cancer Institute–funded program project currently conducted at a National Comprehensive Cancer Center, "Palliative Care for Quality of Life and Symptoms Concerns in Lung Cancer," which serves as a model to promote high-quality care for patients and their families.

Although there has been significant progress made over the past decade in implementing palliative care (PC) in the United States, barriers to its integration for all cancer patients still exist (Institute of Medicine [IOM], 2013). In addition, the population of chronically ill patients with advanced cancers is expanding (Greer, Jackson, Meier, & Temel, 2013). This growth is due to innovational development of combined and targeted chemotherapy regimens (Greer et al., 2013), with the phase between a serious illness and death often extending many years (Ferrell & Coyle, 2010). With chronic terminal care for cancer patients, a high symptom burden is common (Ferrell & Coyle, 2010; IOM, 2013).

The integration of PC into hospital, ambulatory, and community care settings is essential, as patients seek symptom management and high-quality cancer care throughout the trajectory of their illness (Abrahm, 2012; IOM, 2013). Recognized as leading providers of high-quality PC, oncology advanced practitioners (APs) are well positioned to play a key role in integrating PC into their daily practice (McHugh, Arnold, & Buschman, 2012). Palliative care principles utilize a holistic approach to quality-of-life (QOL) concerns of the physical, psychological, social, spiritual, and cultural needs of patients and their families (McHugh, et al., 2012).

## Barriers to Implementing Palliative Care

Many health-care professionals and the general public generally associate PC with hospice or end-of-life care (Ferrell & Coyle, 2010). Within a facility specialized in cancer treatment, such a conversation becomes even more guarded, as a curative focus is primary. A discussion about PC immediately evokes a loss of hope and a sense of futility for both patients and health-care providers (Abrahm, 2012; Ferrell & Coyle, 2010). Misconceptions further complicate the understanding of PC with patients, families, and oncology professionals (Abrahm, 2012; Storey, Fallon, & Smyth, 2011).

In 2011, research conducted by a national poll commissioned by the Centers to Advance Palliative Care (CAPC), with the American Cancer Society (ACS) Cancer Action Network (ACS-CAN), assessed the opinion of both the public and physicians in regard to PC (CAPC, 2011). The findings provided a guide for communicating with consumers and policymakers on the benefits and future direction of PC (CAPC, 2011).

Public opinion data revealed that of 800 adults, aged 18 years old and older, 70% of Americans were not educated on the subject of PC (CAPC, 2011). These findings also revealed that once consumers understood that PC provides relief of symptoms, pain, stress—and that it is appropriate at any stage of serious illness—92% stated they would likely consider it for themselves or their families (CAPC, 2011). Furthermore, of these consumers, 95% also agreed that patients and families should be educated about PC (CAPC, 2011).

The opinion of physicians on PC also revealed key findings in the CAPC report. In fact, the report indicated that physicians may be even less comfortable in discussing PC than patients and caregivers (CAPC, 2011). Participating physicians seemed to equate PC with hospice or end-of-life care and were somewhat opposed to believing otherwise (CAPC, 2011). They also saw PC only as comfort care in the last few weeks or days of life, allowing patients to pass with peace and dignity (CAPC, 2011). According to the CAPC, these findings are significant because they demonstrate a lack of understanding among referring physicians in regard to the impact of PC in providing high-quality cancer care throughout the cancer trajectory.

In addition, a national poll conducted by the National Journal and The Regence Foundation found that 97% of responding physicians confirmed the importance of educating patients and their families about PC (National Journal and The Regence Foundation, 2011). Even though physicians confirmed the importance of educating patients and families, public opinion data validated that patients and families failed to have an understanding of PC due to a lack of communication and education from their providers (CAPC, 2011; Kirch & Brawley, 2012). Additionally, according to the current literature, patients received an inadequate explanation of their treatments and the adverse impact they may have on their future health (IOM, 2013).

In 2013, Greer and colleagues cited data from a national survey of oncologists, revealing that only a minority of these physicians reported they frequently referred patients with cancer to a pain or PC specialist (Bruera & Hui, 2010). Part of the problem may originate with oncologists, not the system (Abrahm, 2012). Some oncologists exhibit what is described as "learned helplessness" from years of practice and lack of effective symptom management training (Abrahm, 2012). Also, some oncologists and APs lack training in handling the communication challenges they may face (Abrahm, 2012). Unless oncologists and the practitioners they train have an opportunity to work with a PC team, they are unlikely to be aware of and knowledgeable about the positive outcome of PC on the quality of care provided to patients at any stage of disease (Abrahm, 2012).

Additional variables may impact the provision of high-quality cancer PC. According to Goldsmith and colleagues (2008), community hospitals (in some cases) serve as the only option for medical care for uninsured patients and geographically isolated communities, and many may lack the resources to provide high-quality care. Changing demographics in the United States, such as the growing number of aging adults and the increasing demand for cancer care, is another concern (IOM, 2013). The oncology workforce may be declining, with fewer professionals able to care for this growing cancer population (IOM, 2013). Moreover, the Centers for Medicare & Medicaid Services, the largest insurer for the elderly, is struggling financially (IOM, 2013). Lastly, the cost of cancer treatments is escalating, making cancer care less affordable for patients and creating greater disparities in patients’ access to high-quality care (IOM, 2013).

## Identifying the Need for PC in Standard Oncologic Care

The IOM released a series of consensus reports entitled Ensuring Quality of Cancer Care (1999); Improving Palliative Care for Cancer (2001); and Delivering High-Quality Cancer Care: Charting a New Course for a System in Crisis (2013), identifying the need to incorporate PC into standard oncologic care. The 2013 consensus report cited that currently approximately 14 million people have been diagnosed with cancer in the United States and that more than 1.6 million new cases are diagnosed each year (IOM, 2013). The report projected that by 2022, there will be 18 million cancer survivors (IOM, 2013). The incidence of cancer is expected to rise to 2.3 million new diagnoses each year (IOM, 2013). Advocacy policy statements have been published by key organizations, concurring that optimal cancer and end-of-life care requires access to state-of-the-art PC rendered by skilled clinicians and supported when necessary by PC experts (Ferris et al., 2009; Bruera & Hui, 2010; National Consensus Project for Quality PC [NCP], 2013).

In addition, Ferris et al. (2009) cited expanding evidence supporting the efficacy of PC in improving patient-reported outcomes such as QOL, depression, and overall survival. Even with supporting evidence of the benefits of PC in cancer care, it is not being implemented into routine oncologic care (Ferris et al., 2009; IOM, 2001, 2013; Temel et al., 2010; Bruera & Hui, 2010; Debono, 2011; Hui et al., 2010; Smith et al., 2012; Storey et al., 2011).

A survey conducted by Hui and colleagues (2010) identified that in all National Cancer Institute(NCI)-designated cancer centers, only 60% had a formal outpatient PC medicine clinic. This number is smaller for non–NCI-designated cancer centers (22%; Hui et al., 2010). Challenges remain because PC as a model of care is inherently diverse: One model may not be feasible in multiple cancer settings or systems (Abernethy & Currow, 2011).

Greer and colleagues (2013) cited data that even in comprehensive cancer centers with abundant resources, oncologists underutilized PC services (Bruera & Hui, 2010). Many of these physicians also were prone to make late referrals to PC medicine over the course of disease (Hui et al., 2010; Temel et al., 2010; Bruera & Hui, 2010).

Since the release of the reports by the IOM and published advocacy statements, it is encouraging to state that progress has been made in the integration of PC (Bruera & Hui, 2010; NCP, 2013; Koczywas et al., 2013). Major hospice and key organizations are working together to integrate PC when caring for patients with cancer, which is recommended by the IOM (Bruera & Hui, 2010; Temel et al., 2010; Koczywas et al., 2013; NCP, 2013).

## A Glimpse at the IOM Recommendations

Recommendations by the IOM for improving the quality of cancer care accounted for the varied nature of cancer care as well as the existing models of high-quality care (IOM, 2013). The goal of the IOM committee recommendations is to provide comprehensive, patient-centered, evidence-based, high-quality cancer care that is accessible and affordable to all in the United States, regardless of where the cancer care is provided (IOM, 2013).

The IOM report included an outline of a conceptual framework (Table 1) to improve the quality of care for patients facing cancer (IOM, 2013). The report urged the entire health-care industry (including all stakeholders) to reevaluate their roles and responsibilities in cancer care and work together to develop a higher-quality cancer care delivery system nationally (IOM, 2013). The report underscored the importance of developing, testing, and disseminating disease-specific models of PC (IOM, 2013), which can be successfully integrated into organizational systems to address the escalating challenges of delivering high-quality care (IOM, 2013; Koczywas et al., 2013). Development of disease-specific PC models may provide patients and families with support mechanisms more relevant to their needs (Gaertner, Wolf, Hallek, Glossman, & Voltz, 2011; IOM, 2013).

**Table 1:**
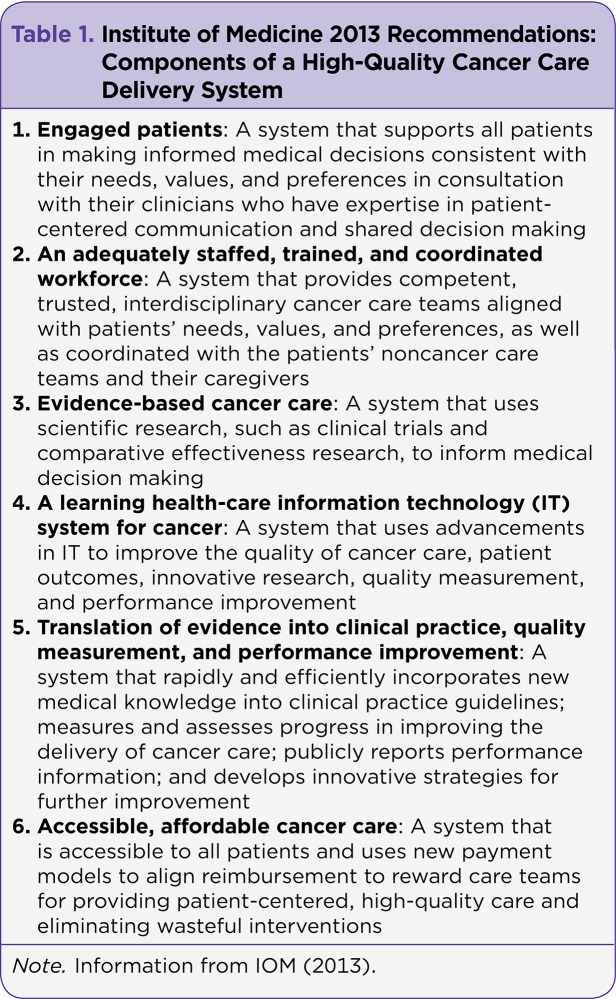
Institute of Medicine 2013 Recommendations: Components of a High-Quality Cancer Care Delivery System

## Creating Clinical Practice Guidelines

The mission of the NCP is to create clinical practice guidelines that improve the quality of PC in the United States (NCP, 2013). Guidelines are specifically intended to promote quality PC, foster consistent and high standards in PC, and encourage continuity of care across clinical settings (NCP, 2013).

The NCP comprised major hospice and key PC organizations and created clinical practice guidelines for quality PC (NCP, 2013). The guidelines described eight core concepts and structures for quality PC (Ferrell & Coyle, 2010; NCP, 2013). The development and revisions of these guidelines were accomplished through a consensus process (Ferrell & Coyle, 2010; NCP, 2013). The clinical practice guidelines for quality PC set high expectations for excellence, not basic competence for existing programs (Ferrell & Coyle, 2010; NCP, 2013).

In 2006, the National Quality Forum (NQF) endorsed the guidelines and established initial areas within which to develop outcome measures for PC programs (NCP, 2013). In 2008, the National Priorities Partnership, a consortium of US Health Care Organizations working together with the NQF, identified PC as one of six top priorities for improving the US health-care system (NCP, 2013).

Revisions of the guidelines continued in 2009 and again in 2013, which reflected ongoing collaboration to refine core concepts and structures for quality PC (NCP, 2013). The new guidelines (Table 2) focus on psychosocial and spiritual care, with sensitivity to patient and caregiver needs, preferences, values, beliefs, and culture (NCP, 2013). The focus is on quality and equitable access to PC services and rests on the values of assessment, information sharing, decision-making, care planning, and continuity of care across all health-care settings (Ferrell & Coyle, 2010; NCP, 2013).

**Table 2:**
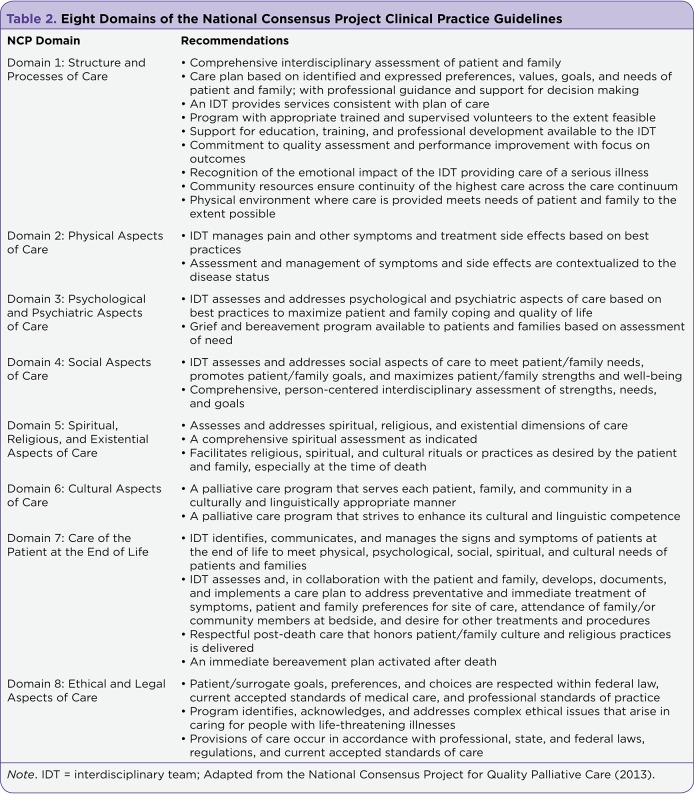
Eight Domains of the National Consensus Project Clinical Practice Guidelines

## An Interdisciplinary Model of PC in Lung Cancer

According to the ACS, in 2014, it is estimated that there will be approximately 224,000 new cases of lung cancer and about 159,000 deaths from lung cancer, accounting for about 27% of all cancer deaths (ACS, 2014). As in other cancer settings, challenges and barriers exist in the integration of models of PC into routine oncologic care in lung cancer (Koczywas et al., 2013).

Studies have shown that patients with metastatic non–small cell lung cancer (NSCLC) benefit from early PC (Temel et al., 2010, 2011). Metastatic lung cancer remains an incurable disease, causing significant morbidity and a high symptom burden (Ferrell, Koczywas, Grannis, & Harrington, 2011; Temel et al., 2010, 2011). There is no doubt that our current cancer care delivery system remains deficient, often failing to meet the needs of patients facing lung cancer and their families (IOM, 2013; Ferrell et al., 2011; Temel et al., 2010, 2011).

Along with the stigma of having lung cancer, patients experience complex symptoms affecting specific facets of QOL (physical, psychological, social, spiritual), as do family caregivers (Ferrell et al., 2011; Temel et al., 2010, 2011). Improved, comprehensive models of PC implemented in lung cancer care are needed. The development of a comprehensive model of PC, which focuses on minimizing the expected symptom burden related to terminal illness while maximizing overall QOL, is currently being conducted at a National Comprehensive Cancer Network–designated urban hospital with the use of an oncology AP (Koczywas et al., 2013).

The Palliative Care for Quality of Life and Symptoms Concerns in Lung Cancer project is an NCI-funded program project grant being conducted at the City of Hope in Duarte, California. The primary purpose of this 5-year study is to compare usual care with an interdisciplinary PC educational intervention delivered by oncology APs for patients with NSCLC (Koczywas et al., 2013). Findings from the usual-care phase of this study informed the development of the interdisciplinary PC intervention (Koczywas et al., 2013).

Three simultaneous projects are included within the program project. Project 1 focuses on early-stage lung cancer and provides a model of integrating PC throughout the trajectory of disease. Project 2 focuses on late-stage lung cancer, a population that has decreased survival, a high symptom burden, and QOL concerns. Project 3 focuses on family caregivers of patients with lung cancer (Ferrell et al., 2011; Koczywas et al., 2013). This model of care was developed based on extensive pilot work (Borneman et al., 2008; Ferrell et al., 2011; Koczywas et al., 2013; Podnos, Borneman, Koczywas, Uman, & Ferrell, 2007).

A comprehensive assessment of QOL concerns of both patients and family caregivers prior to treatment initiation begins this process of care (Ferrell et al., 2011; Koczywas et al., 2013). Quality-of-life assessment focuses on four QOL domains: physical, psychological, social, and spiritual well-being (Ferrell et al., 2011; Koczywas et al., 2013). Following the comprehensive QOL assessment, an interdisciplinary team conference is scheduled and initiated. The team includes the patients’ treating physician(s); an oncology AP involved in patient care; as well as supportive care experts such as PC experts, social workers, psychologists, spiritual counselors, pulmonary rehabilitation specialists, geriatric oncologists, and dieticians (Ferrell et al., 2011; Koczywas et al., 2013).

The interdisciplinary team discusses the patient’s QOL assessment, and a care plan is produced to address each of the issues (Ferrell et al., 2011; Koczywas et al., 2013). The AP coordinates the care, based on the recommendations of the interdisciplinary team and the patient’s goals of care (Koczywas et al., 2013). This plan includes patient and family caregiver education provided by the AP, support from team members, and referrals to supportive care services (Ferrell et al., 2011; Koczywas et al., 2013). Patient and family caregiver outcomes measured include QOL; functional status; support services utilization; distress; resource utilization; and family caregiver perception of self-care, caregiver burden, and skills preparedness (Ferrell et al., 2011; Koczywas et al., 2013).

Consistent with the 2013 IOM recommendations on PC, researchers at the City of Hope believe that PC, including symptom management and attention to the QOL concerns of both patients and family caregivers, should be addressed throughout the trajectory of lung cancer (Ferrell et al., 2011; Koczywas et al., 2013). The PC model discussed in this study easily allows oncology APs to implement key PC principles. APs can execute assessment practices using a holistic approach, focusing on QOL domains with attention to supporting caregiver needs.

## Implications for Advanced Practitioners

Oncology APs who specialize in PC nursing are well positioned to lead the way in providing high-quality cancer care, as we seek advanced PC education and develop clinical expertise (McHugh, et al., 2012). Advanced practitioners play an essential role in educating patients as to how PC can be a critical part of treatment regardless of curability, in addition to communicating and clarifying patients’ understanding of their prognosis (Greer et al., 2013; Abrahm, 2012; Bruera & Hui, 2010; Bruera & Yennurajalingam, 2012; Ferrell & Coyle, 2010; IOM, 2013; Kirch & Brawley, 2012; Storey et al., 2011). Proper education empowers patients and their families to make informed decisions about treatment options (Greer et al., 2013; Abrahm, 2012; Bruera & Hui, 2010; Bruera & Yennurajalingam, 2012; Ferrell & Coyle, 2010; IOM, 2013; Kirch & Brawley, 2012; Storey et al., 2011). A disconnect in communication between patients and clinicians impedes treatment decision-making, deprives patients of hope, creates a sense of loss of control, and silences patients’ voices (Greer et al., 2013; Wittenberg-Lyles, Goldsmith, Ferrell, & Ragan, 2013).

Advanced practitioners play a key role in integrating PC while caring for chronically and terminally ill patients. This step requires advanced educational preparation and development of clinical expertise. In addition, effective and thoughtful communication skills are required. Effective communication empowers patients and families and strengthens the patient/nurse and family caregiver/nurse relationship (Wittenberg-Lyles et al., 2013).

Oncology APs must educate nurses across all specialty areas on key PC principles and the importance of providing specialized care. PC principles are integral to the scope and standards of professional nursing and all specialty areas of nursing practice (McHugh, et al., 2012). Oncology APs can serve as the leading providers of PC in providing high-quality cancer care for all cancer patients and their families.
